# Systematic assessment of cervical cancer initiation and progression uncovers genetic panels for deep learning-based early diagnosis and proposes novel diagnostic and prognostic biomarkers

**DOI:** 10.18632/oncotarget.22689

**Published:** 2017-11-25

**Authors:** Nguyen Phuoc Long, Kyung Hee Jung, Sang Jun Yoon, Nguyen Hoang Anh, Tran Diem Nghi, Yun Pyo Kang, Hong Hua Yan, Jung Eun Min, Soon-Sun Hong, Sung Won Kwon

**Affiliations:** ^1^ College of Pharmacy, Seoul National University, Seoul 08826, Korea; ^2^ Department of Drug Development, College of Medicine, Inha University, Incheon 22212, Korea; ^3^ School of Medicine, Vietnam National University, Ho Chi Minh 70000, Vietnam; ^4^ Research Institute of Pharmaceutical Sciences, Seoul National University, Seoul 08826, Korea

**Keywords:** cervical cancer, transcriptomics, deep learning, meta-analysis, survival analysis

## Abstract

Although many outstanding achievements in the management of cervical cancer (CxCa) have obtained, it still imposes a major burden which has prompted scientists to discover and validate new CxCa biomarkers to improve the diagnostic and prognostic assessment of CxCa. In this study, eight different gene expression data sets containing 202 cancer, 115 cervical intraepithelial neoplasia (CIN), and 105 normal samples were utilized for an integrative systems biology assessment in a multi-stage carcinogenesis manner. Deep learning-based diagnostic models were established based on the genetic panels of intrinsic genes of cervical carcinogenesis as well as on the unbiased variable selection approach. Survival analysis was also conducted to explore the potential biomarker candidates for prognostic assessment. Our results showed that cell cycle, RNA transport, mRNA surveillance, and one carbon pool by folate were the key regulatory mechanisms involved in the initiation, progression, and metastasis of CxCa. Various genetic panels combined with machine learning algorithms successfully differentiated CxCa from CIN and normalcy in cross-study normalized data sets. In particular, the 168-gene deep learning model for the differentiation of cancer from normalcy achieved an externally validated accuracy of 97.96% (99.01% sensitivity and 95.65% specificity). Survival analysis revealed that ZNF281 and EPHB6 were the two most promising prognostic genetic markers for CxCa among others. Our findings open new opportunities to enhance current understanding of the characteristics of CxCa pathobiology. In addition, the combination of transcriptomics-based signatures and deep learning classification may become an important approach to improve CxCa diagnosis and management in clinical practice.

## INTRODUCTION

Cervical cancer (CxCa) is currently the fourth most commonly diagnosed form of cancer and one of the leading causes of cancer-related mortality in women around the world [1, 2]. Human papillomavirus (HPV) has been involved in the initiation and progression of approximately 99% of cervical tumors, in which HPV 16 and HPV 18 contribute approximately 70% [3–5]. In recent decades, thanks to vaccination and screening tests, CxCa-associated death rate has declined significantly. However, the great burden of CxCa still remains a critical issue, especially in developing countries [6]. Current treatment strategies have certain limitations and induce a wide range of side effects on CxCa patients [7, 8]. Moreover, patients with cervical precancerous lesions have a five-year survival rate of nearly 100% [9], encouraging scientists to focus on studying the early-stage of CxCa carcinogenesis.

For a long time, microscopic biopsy image analysis has been the backbone of screening and diagnostic processes of CxCa [10]. However, this technique may not be a reliable measure since it is based on subjective observations. Although novel biomarkers have been increasingly studied to complement the limitations of standard cytological evaluations, the lack of suitable biomarkers for monitoring cancer progression of cervical dysplasia has remained a challenging issue and usually results in the use of improper treatments [11, 12]. These challenges may stem partially from the lack of profound understanding of the mechanisms of cancer initiation and progression [13]. In addition, the regulations of many CxCa driver genes remain comparatively unknown. Clinically accurate biomarkers or gene expression signatures as well as more systematic approaches, such as supervised learning meta-analysis based frameworks, are still needed. Eventually, it is important to place more effort on discovering and validating potential biomarker candidates, individuals or panels, to improve diagnostic and prognostic assessment.

Meta-analysis and cross-study normalization are two fundamental approaches to integrating data from different microarray data sets. Meta-analysis combines data at the “late stage”, while cross-study normalization combines data at the “early stage” [14]. Cross-study transformation and normalization merges data from multiple microarray studies of a common organism and phenotype, removes non-biological differences, also known as batch effects, and then increases the sample size and retains high prediction accuracy of the machine learning-based class assignment analysis [15]. In addition to cross-study normalization, meta-analysis provides a flexible and powerful approach by integrating different microarray data sets and platforms to increase the statistical power, reliability, and generalizability of the results. Therefore, the meta-analysis of genome-wide gene expression to identify corresponding molecular mechanisms of cervical carcinogenesis has the potential to improve the prediction of risk, diagnostic decision, prognostic evaluation, and treatment. This method also generates a reasonably complete picture of differentially expressed genes and pathways involved in cancer [16].

In the current study, we integrated available Affymetrix-based microarray data of CxCa patients and conducted a comprehensive meta-analysis on differentially expressed genes, pathway enrichment analysis, and network analysis. Genetic panels that were associated with the initiation and progression of CxCa and their impacts on CxCa diagnosis were proposed and examined. The diagnostic analysis was conducted using a state-of-the-art feed forward deep learning technique. The impact of individual genes on the survival of CxCa patients was further examined using survival analysis of available data sources. Finally, novel candidates were introduced for further investigations of CxCa pathobiology.

## RESULTS

### Gene expression microarray data set identification and selection

**Figure 1:**
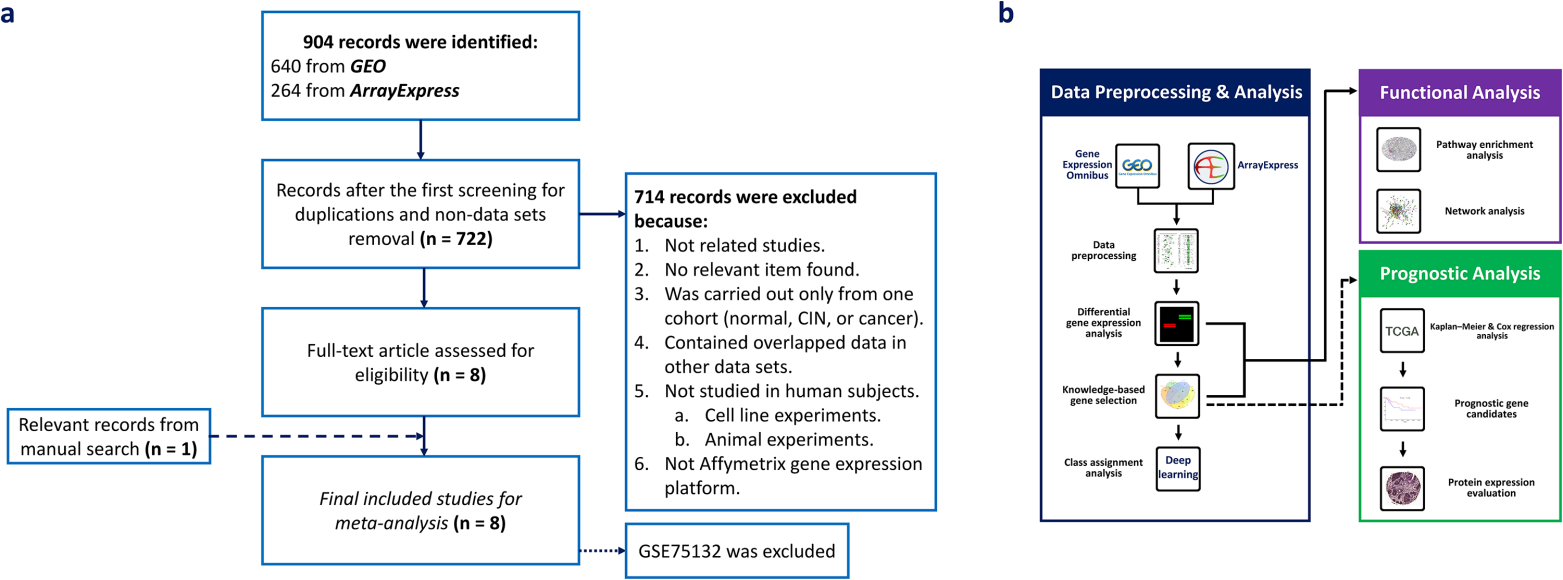
Overview of the study flow **(a)** Flow diagram for data set selection. **(b)** The workflow of data processing and analysis.

The overall search workflow, data processing, and data analysis of this study are presented in Figure 1. After searching in Gene Expression Omnibus (GEO) and ArrayExpress, we retrieved 640 and 264 records, respectively. We then removed 182 duplicate records between these two databases and obtained a final total of 722 records for screening. Seven hundred fourteen records were excluded because they met one or more predefined exclusion criteria. It is worth noting that GSE39001 and 29570 were excluded because they overlapped with GSE52903. We assessed full-text articles of the remaining eight data sets to evaluate the eligibility of each data set. As a result, GSE75132 was excluded from further investigation because the study combined CIN 3 and cancer samples into one group (CIN 3+). We also conducted a manual search of the reference lists of included data sets, and GSE42764 was found to qualify our inclusion criteria. Therefore, we finally assessed eight data sets to carry out the meta-analysis.

Among the eight data sets, we classified and arranged the samples into three main groups: Cancer versus Normalcy, CIN versus Normalcy, and Cancer versus CIN, which contained eight data sets, three data sets, and two data sets, respectively. Cancer versus Normalcy implied the “overall” alteration in genome-wide gene expression, and we observed multistep carcinogenesis mainly by adding CIN versus Normalcy and Cancer versus CIN groups. Cancer versus Normalcy was the group containing the largest number of samples, 93 normal and 202 cancer samples, followed by CIN versus Normalcy group, containing 46 normal samples and 115 CIN samples. The number of samples of Cancer versus CIN group was smaller than that of the two above groups, containing 83 CIN and 49 cancer samples, respectively. The characteristics of the included data sets are shown in Table 1. Finally, the gene expression of the high-grade squamous intraepithelial lesion (HSIL) versus low-grade squamous intraepithelial lesion (LSIL) in CIN group was also investigated.

**Table 1:** Microarray data sets in the meta-analysis of CxCa

Comparison	Author	Data set	Year	Platform^1^	Country	Participants		
***Cancer versus Normalcy***						Normalcy	CIN(s)	Cancer
	Martinez IM *et al.* [69]	GSE52903	2015	1.0 ST	Mexico	17	-	55
	den Boon JA *et al.* [70]	GSE63514	2015	U133 Plus 2.0	USA	24	-	28
	Polyzos A *et al.* [71]	GSE63678	2015	U133A 2.0	Greece	5	-	5
	Yan R *et al.*	GSE42764	2014	U133 Plus 2.0	Canada	2	-	12
	Karagavriilidou K *et al.*^2^[72]	GSE27678	2013	U133 Plus 2.0	UK	3	-	28
	Murty VV *et al.* [73]	GSE9750	2008	U133A	USA	24	-	33
	Zhai Y *et al.* [74]	GSE7803	2007	U133A	USA	10	-	21
	Pyeon D *et al.* [28]	GSE6791	2007	U133 Plus 2.0	USA	8	-	20
***CIN***^3^ ***versus Normalcy***								
	den Boon JA *et al.*	GSE63514	2015	U133 Plus 2.0	USA	24	76	-
	Karagavriilidou K *et al.*^2^	GSE27678	2013	U133A	UK	12	32	-
	Zhai Y *et al.*	GSE7803	2007	U133A	USA	10	7	-
***Cancer versus CIN***								
	den Boon JA *et al.*	GSE63514	2015	U133 Plus 2.0	USA	-	76	28
	Zhai Y *et al.*	GSE7803	2007	U133A	USA	-	7	21

^1^ All included data sets belong to Affymetrix platform.

^2^ GSE27678 contains two different platform (U133 Plus 2.0 and U133A).

^3^ Cervical intraepithelial neoplasia or cervical dysplasia.

### Meta-analysis on differentially expressed genes in cervical cancer

We identified 5,679 significantly differentially expressed genes (DE genes) in the meta-analysis across eight data sets from Cancer versus Normalcy group. Among the DE genes, 2,877 genes were upregulated (125 genes having the combined effect size > 2) and 2,802 genes were downregulated (46 genes having the combined effect size < -2) in terms of cancer patients versus normal controls. CDKN2A (3.81), DTL (3.35), MCM2 (3.34), ECT2 (3.10), RFC4 (3.03), CDC7 (2.99), MELK (2.97), PRC1 (2.96), TOPBP1 (2.93), and STIL (2.92) were the 10 genes that had the highest combined effect sizes. On the other hand, CRNN (-3.05), ENDOU (-3.03), UPK1A (-3.01), EDN3 (-2.79), SLC27A6 (-3.08), CRISP3 (-2.78), ALOX12 (-2.76), AR (-2.73), HOPX (-2.73), and MAL (-2.61) were the 10 genes that had the lowest combined effect sizes. The total number of significantly DE genes of CIN versus Normalcy and Cancer versus CIN groups were 1,989 (1,153 upregulated, two genes with combined effect size > 2 and 836 downregulated, one gene with combined effect size < -2) and 1,986 (994 upregulated, no gene with combined effect size > 2 and 992 downregulated, two genes with combined effect size < -2), respectively. The list of those DE genes is provided in Supplementary File 1.

Since a network-based analysis approach can be applied for discovering biomarker candidates and potential therapeutic targets [17], we conducted a network-based meta-analysis to identify the most potential hub genes that may be considered as important genes in CxCa pathobiology. According to the analysis, HDAC1 was considered as the most potential hub gene for Cancer versus Normalcy group. Its degree of centrality (DC) and betweenness centrality (BC) were 209 and 582,340.1, respectively. The other distinguished genes of the network included EP300 (DC = 184, BC = 748,497.9), CDK2 (DC = 170, BC = 374,919.2), and MAGOH (DC = 168, BC = 533,497.6). The prominent hub genes in CIN versus Normalcy group included HDAC1 (DC = 209, BC = 570,436.2) and MAGOH (DC = 168, BC = 503,691.5), among others. Finally, CREBBP (DC = 190, BC = 512,840.8) and CDK2 (DC = 170, BC = 409,517.7) were recorded as the most potential hub genes in Cancer versus CIN group, whereas HDAC1 was a relatively low connected gene in this group (DC = 18, BC = 30,041.4). The size of the network, and DC and BC values of other genes in the network analysis can be found in Supplementary File 2.

### Pathway enrichment analysis for detecting biologically meaningful processes of CxCa carcinogenesis

To further identify the biologically meaningful pathways that were involved in CxCa from the DE genes, we performed pathway enrichment analysis. The analysis was performed for upregulated and downregulated DE genes separately for each comparison group. The significantly enriched pathways were considered if they met the qualification criteria of *P*-value < 0.05 and false discovery rate (FDR) < 0.2. In the upregulated gene groups, Cancer versus Normalcy group exhibited 21 significantly enriched pathways. The CIN versus Normalcy group consisted of 10 significantly enriched pathways, while only four enriched pathways were found in Cancer versus CIN group. Interestingly, cell cycle appeared to be the common and most significantly enriched pathway in all comparison groups. Notably, the cell cycle genes of the three different comparison groups were not completely overlapping. This result may come from the heterogeneity in different stages of the disease. In addition, RNA transport and mRNA surveillance pathways were two others common significant pathways among the three groups. Although one carbon pool by folate, pyrimidine metabolism, and p53 signaling pathways were only significant in Cancer versus Normalcy and CIN versus Normalcy comparison groups, these dominant pathways may also be important in CxCa carcinogenesis. Finally, when considering the aberrantly expressed genes in CIN 2 & CIN 3 (HSIL) relative to CIN 1 (LSIL) using available data from GSE27678 and GSE63514, we discovered the upregulation of cell cycle genes (CDC6, CDC7, CDK4, CDC20, CDKN2C, MCM6, MCM5, MCM3, MCM2, PRKDC, BUB3, and PCNA) and one carbon pool by folate-related genes (TYMS, SHMT2, and DHFR). This observation, once again, emphasized the important roles of the cell cycle and one carbon metabolism in CxCa initiation and progression. It is important to note that there were many genes that are known to be associated with cancer, as shown in the “pathways in cancer” enriched pathway. In the same pattern, we investigated enriched pathways from downregulated genes in the three groups. We obtained 15 enriched pathways in Cancer versus Normalcy group, including several well-known pathways, such as the calcium signaling pathway, Wnt signaling pathway, Hedgehog signaling pathway, and linoleic acid metabolism, as well as gap junction and focal adhesion pathways. Surprisingly, our results indicated a poor number of enriched pathways in CIN versus Normalcy and Cancer versus CIN groups. Furthermore, the Wnt signaling pathway and Hedgehog signaling pathway seem enriched in CIN versus Normalcy and Cancer versus CIN groups although its FDR was greater than 0.2. The mammalian circadian rhythm pathway was the only pathway significantly enriched in CIN versus Normalcy group while the amoebiasis pathway was the unique pathway in Cancer versus CIN group. Table 2 and Supplementary File 3a and 3b show major enriched pathways and their corresponding genes in corresponding comparison groups.

**Table 2:** Some representative up- and downregulated pathways in the differential gene expression meta-analysis

	Pathway	Number of gene	Gene symbols	P-value	FDR
Upregulated pathway					
**Cancer versus Normal**	Cell cycle	36	CDC6, MCM5, MCM3, ORC2, CDC7, RBL1, YWHAH, CCNB1, CDK4, SMAD2, CCNE1, PCNA, CDKN2C, E2F3, EP300, MCM6, MCM2, STAG2, CDK2, CCNA2, CCNB2, MAD2L1, RAD21, CDC23, ORC5, CDC25A, CDC20, CDC25C, SMC1A, TFDP2, PTTG1, E2F1, SMC3, BUB3, HDAC1, HDAC2	1.43E-13	3.10E-11
	RNA transport	26	NXT2, NUP107, NUP155, EIF2S3, NXT1, EIF2S1, NUPL2, NUP133, NUP153, UPF1, EIF2B2, NUP205, PAIP1, GEMIN2, NUP93, SUMO4, MAGOHB, NUP43, UPF2, MAGOH, EIF2S2, NCBP1, NUP160, NUP58, XPO1, EIF2B1	8.69E-7	9.43E-5
	mRNA surveillance pathway	18	PABPN1, NXT2, NXT1, UPF1, PPP2R3A, PELO, NUDT21, MAGOHB, PPP2R5E, CPSF7, UPF2, SMG5, MAGOH, CSTF2, NCBP1, CPSF6, GSPT1, SMG1	1.81E-5	1.31E-3
**CIN versus Normal**	Cell cycle	31	CDC6, MCM5, MCM3, CDC7, CCNB1, HDAC2, CDK4, CCNE1, CDKN2C, E2F3, MCM6, MCM2, CCNA2, CCNB2, MAD2L1, RAD21, ORC5, CDC25A, CDC20, PRKDC, CDC25C, SMC1A, PTTG1, SMC3, BUB3, HDAC1, RBL1, PCNA, STAG1, CDC23, TFDP2	1.21E-20	2.62E-18
	One carbon pool by folate	6	TYMS, SHMT2, MTR, GART, MTHFD2, DHFR	1.61E-5	1.75E-3
	RNA transport	14	NXT2, NUP107, NUP155, NUPL2, NUP133, PAIP1, MAGOHB, NUP43, UPF2, MAGOH, EIF2S2, NCBP1, NUP160, XPO1	3.02E-5	2.18E-3
**Cancer versus CIN**	Cell cycle	14	MCM3, ORC2, CREBBP, E2F3, CDK2, MAD2L1, RAD21, PRKDC, E2F1, BUB3, ABL1, RBL1, CDC23, WEE1	8.60E-7	1.87E-4
	mRNA surveillance pathway	9	MAGOHB, PPP2R5E, CPSF7, UPF2, NCBP1, CPSF6, GSPT1, PPP2R2D, SMG1	1.10E-4	1.20E-2
	Pathways in cancer	16	CREBBP, E2F3, CBL, CDK2, TCF7L1, FZD7, MAP2K1, ITGB1, CXCL8, WNT11, E2F1, CRKL, ABL1, STK4, ETS1, PIAS2	2.16E-3	1.42E-1
**Down-regulated pathway**					
**Cancer versus Normal**	Phototransduction	8	RCVRN, GUCA1B, GNAT1, GNAT2, GUCY2D, RHO, CALML3, ARRB1	2.37E-4	3.18E-2
	Focal adhesion	26	PIK3R2, HGF, VTN, CCND1, PDGFRA, CCND2, VWF, PDGFRB, MAPK3, KDR, SHC2, COL5A3, ITGB5, VEGFD, ILK, PARVA, CAV1, CAV3, ACTN2, JUN, SHC3, ITGA8, PDGFD, RELN, LAMA2, ITGA7	2.93E-4	3.18E-2
	Calcium signaling pathway	23	BDKRB1, PDGFRA, HTR2B, PDGFRB, HTR5A, TACR3, TACR1, ADRB2, CALML3, ATP2B2, GNAL, CACNA1H, CCKBR, ERBB4, ADCY2, TNNC2, GNA14, EDNRB, HTR2A, ADRA1D, GRPR, ADRA1A, ITPR2	6.62E-4	4.79E-2
**CIN versus Normal**	Circadian rhythm - mammal	4	CLOCK, PER1, NPAS2, CNSK1E	6.31E-4	1.37E-1
**Cancer versus CIN**	Amoebiasis	7	HSPB1, GNA15, SERPINB3, IL1R2, GNAL, SERPINB4, RELA	4.45E-5	9.66E-3

### Functional analysis of individual genes that are consistently up- and downregulated

The total number of significantly upregulated genes from the meta-analysis of Cancer versus Normalcy group was 2,877, that of CIN versus Normalcy group was 1,153, and that of Cancer versus CIN group was 994. Using the filter provided by InteractiVenn [18], we obtained 248 overlapping DE genes, which were always upregulated in more aggressive subgroups (i.e., Cancer subgroup in Cancer versus Normalcy group, CIN in CIN versus Normalcy group, and Cancer in Cancer versus CIN group). The total number of significantly downregulated genes of Cancer versus Normalcy, CIN versus Normalcy, and Cancer versus CIN groups were 2,802, 836, and 992, respectively. Using the same approach, 122 overlapped genes were found to be always downregulated in more aggressive subgroups.

**Figure 2:**
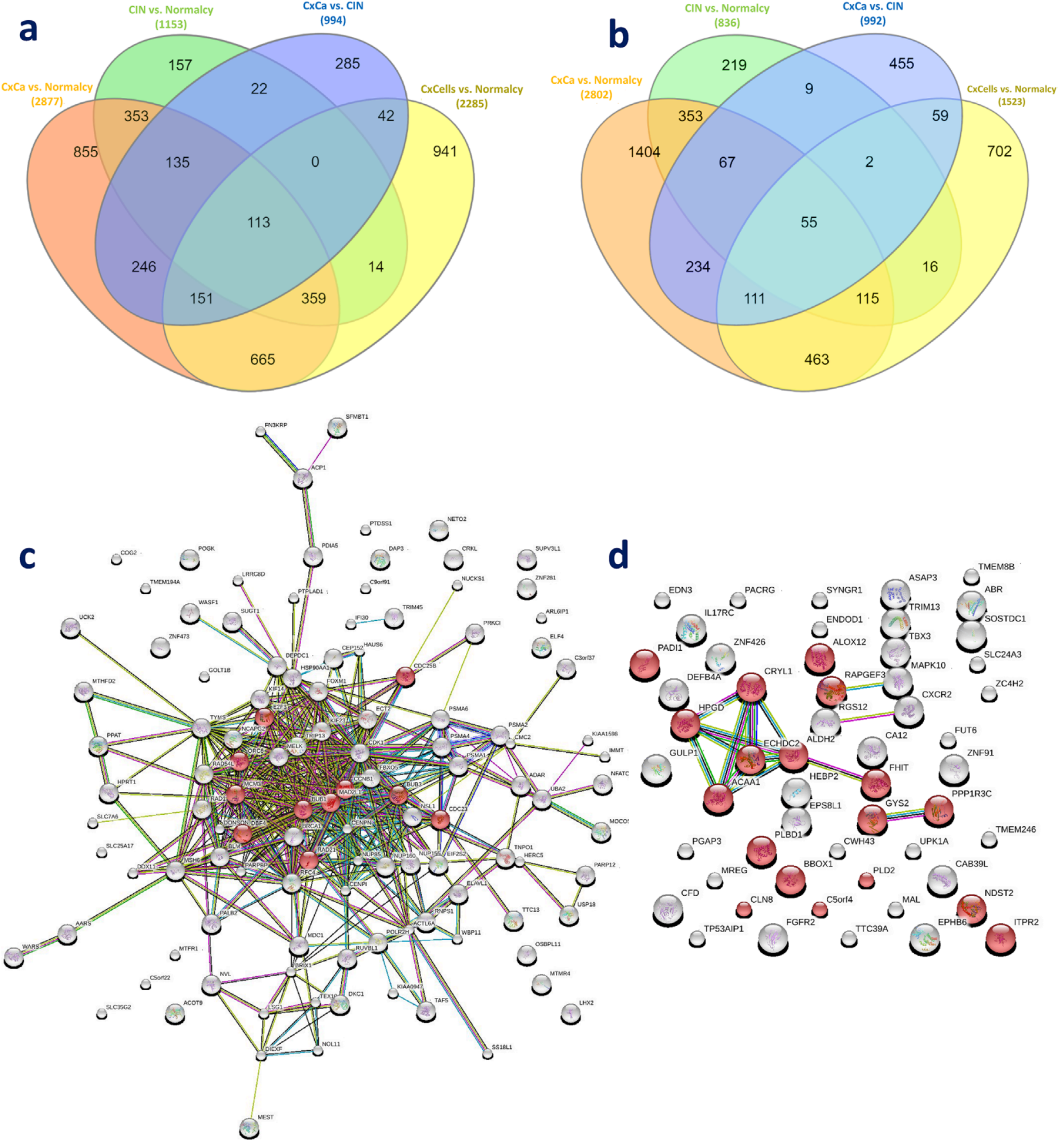
Genetic panel of 168 consistently dysregulated genes **(a)** Overlapping genes in the consistently upregulated group. **(b)** Overlapping genes in the consistently downregulated group. **(c)** Protein-protein network of 113 consistently upregulated genes. **(d)** Protein-protein network of 55 consistently downregulated genes. Red, green, blue, purple, yellow, light blue, and black lines indicate the presence of fusion, neighborhood, co-occurrence, experimental, text mining, database, and co-expression evidence, respectively.

To reduce “false positive” candidates and obtain a more reliable list of up- and downregulated candidate genes, we performed an additional validation for the above overlapping DE genes and DE genes from the meta-analysis of two independent data sets. In this validation, we utilized the DE genes in the comparison between 10 CxCa cell lines (N = 21) and normal tissue from two data sets (GSE29216 and GSE9750) in which the DE genes were identified using the same statistical approach. Finally, 113 upregulated genes were confirmed to be consistently upregulated (Figure 2a). Similarly, 55 consistently downregulated genes were also observed (Figure 2b). Those genes may be considered the intrinsic genetic characteristics for determining cervical carcinogenesis, and the panel can be seen in Table 3.

**Table 3:** The genetic panel for deep learning classification adapted from consistently upregulated and downregulated genes

	Number of gene	Gene symbols
**Upregulated genes**	113	FOXM1, RFC4, ORC6, NCAPG2, KIF23, TRIP13, CENPN, KIF14, TYMS, AARS, DONSON, E2F3, RNPS1, BRIX1, TMEM194A, MELK, MCM3, MOCOS, BUB1, ECT2, CDC25B, DEPDC1, FBXO5, POLR2H, RAD1, CDK1, RAD54L, BUB3, SUPV3L1, DBF4, NETO2, CENPI, SLC7A6, CMC2, MEST, BLM, ZNF281, ACOT9, MAD2L1, DDX11, PDIA5, ELAVL1, NUP155, RUVBL1, WARS, SS18L1, MTHFD2, LRRC8D, MSH6, IMMT, LHX2, RAD21, EIF2S2, MDC1, NUP85, C5orf22, CDC23, UCK2, PTDSS1, UBA2, ZNF473, BRCA1, TMEM22, ADAR, NUP160, ACP1, WBP11, DIEXF, C9orf91, NVL, PTPLAD1, C3orf37, WASF1, HPRT1, ACTL6A, PPAT, DKC1, POGK, MTFR1, ELF4, HAUS6, TEX10, USP18, PRKCI, TNPO1, ARL6IP1, KIAA1598, NFATC2IP, KIAA0947, PARP12, NUCKS1, PARPBP, TAF5, CRKL, GOLT1B, CEP152, SLC25A17, HSP90AA1, HERC5, NSL1, FN3KRP, IFI30, LSG1, PALB2, MTMR4, PSMA6, SFMBT1, TTC13, DAP3, TRIM45, OSBPL11, COG2, NOL11
**Downregulated genes**	55	SYNGR1, CFD, C9orf125, TTC39A, BBOX1, CXCR2, CRYL1, HPGD, HEBP2, MAL, FHIT, EDN3, NDST2, ABR, UPK1A, SOSTDC1, ITPR2, CAB39L, ALOX12, FUT6, TP53AIP1, CD24, MREG, FGFR2, PLD2, EPHB6, ACAA1, CWH43, CA12, ZNF91, IL17RC, TBX3, RAPGEF3, PACRG, ECHDC2, ZC4H2, ASAP3, EPS8L1, ZNF426, ALDH2, C5orf4, MAPK10, SLC24A3, GYS2, PPP1R3C, PADI1, DEFB4A, RGS12, ENDOD1, GULP1, TMEM8B, PGAP3, TRIM13, CLN8, PLBD1

The protein-protein network of consistently upregulated genes as well as known and predicted interactions between the nodes curated by STRING are shown in Figure 2c (113 nodes and 401 edges). There was a strong connection of known and predicted interactions arising from most proteins in the network. In addition, the top 10 enriched GO biological processes and the first enriched KEGG pathway were closely associated with cell cycle. Enriched GO terms were mitotic cell cycle (GO:0000278), nuclear division (GO:0000280), and DNA repair (GO0006281), among others. Ten cell cycle related proteins annotated in KEGG were CDC25B, DBF4, E2F3, ORC6, MCM3, BUB1, RAD21, MAD2L1, CDC23, and BUB3. This observation further emphasized the importance of the cell cycle in CxCa carcinogenesis. Another enriched KEGG pathway was progesterone-mediated oocyte maturation. The protein-protein network of consistently downregulated genes was simpler than that of upregulated genes (54 nodes and 14 edges). HPGD, CRYL1, ECHDC2, ACAA1, ALDH2, and FHIT were strongly connected, and these proteins are associated with metabolic processes (Figure 2d). Small molecule metabolic process (GO:0044281), cellular lipid metabolic process (GO:0044255), single-organism biosynthetic process (GO:0044711), and single-organism cellular process (GO:0044763) were enriched, which demonstrated that there might be a decrease of some metabolic processes, especially lipid metabolism, in CxCa initiation and progression.

### Text mining analysis for the interpretation of the selected genetic panel

To determine whether our obtained candidates were frequently reported together by previous cancer-related studies, we utilized 113 upregulated genes and 55 downregulated genes as inputs for the CCancer text mining database. For upregulated genes, our input list was significantly enriched in previous cancer-related studies, illustrated by the fact that many genes on the list were reported to be significantly associated with several cancers, including CxCa (*P*-value < 0.05). Twenty genes were reported in two previous investigations on CxCa. Their corresponding functions were extracted, and known functions in CxCa were reviewed (Table 4). BUB1, DBF4, DKC1, LSG1, NUP155, POLR2H, and ZNF473 are novel targets for further studies since their roles in CxCa initiation and progression are unknown. On the other hand, we did not achieve any significantly downregulated genes that were associated with CxCa. In terms of other tumors, the text mining results indicated that our provided upregulated genes were also correlated with, but not limited to, breast, skin, colorectal, thyroid, ovarian cancer, lymphoma, myeloid leukemia, neuroblastoma, and retinoblastoma. Contrarily, our downregulated genes were associated with melanoma and “wound healing in the elderly”. The detailed information of text mining results and the matched genes for each condition can be found in Supplementary File 4. These results may provide better insights into the essential molecular mechanisms of not only CxCa but also multi-cancer interactions.

**Table 4:** Reported genes in the two previous literatures on CxCa and their related functions

Article	Gene code	Full name	Effect sizes			Function related to cervical cancer
			Cancer-Normalcy	CIN-Normalcy	Cancer-CIN	
	**BRCA1**	BRCA1, DNA repair associated	2.04	1.42	0.66	Hypermethylation of the BRCA1 promoter was observed in advanced stage invasive cervical cancer patients [76]. Another study reported that BRCA1 promoter methylation may be related to worse prognosis since patients carrying this mutation failed to respond to the treatment [77].
	**BUB1**	BUB1 mitotic checkpoint serine/threonine kinase	1.71	0.81	0.78	BUB1 has not been described to be associated with cervical cancer.
	**CDK1**	Cyclin dependent kinase 1	2.42	1.52	0.65	Cyclin B1, a regulatory subunit of CDK1 and a crucial protein for the transition from G2 phase to mitosis of the cell cycle, is found to be overexpressed in invasive cervical cancer cells [78]. The Human Papillomavirus E6 oncoprotein abolishes cell cycle checkpoints, inducing polyploidy, an early step in the carcinogenesis of cervical cancer. And the upregulation of CDK1 was observed in this process [79].
	**DBF4**	DBF4 zinc finger	1.99	1.34	0.91	DBF4 has not been described to be associated with cervical cancer.
	**ECT2**	Epithelial cell transforming 2	3.10	1.46	1.40	The high expression of ECT2 in the region 3q may be implicated in cervical oncogenesis [80].
**Identification of a proliferation gene cluster associated with HPV E6/|E7 expression level and viral DNA load in invasive cervical carcinoma** **[75]**	**FBXO5**	F-box protein 5	1.89	1.35	0.75	A study suggested that the differential regulation of miR-654-3p on FBXO5 may enforce cell cycle progression and cause genomic instability in CIN III stage [81].
	**FOXM1**	Forkhead box M1	2.78	1.25	1.19	High levels of FOXM1 expression were observed in cervical cancer. Its overexpression was correlated with tumor aggressiveness and the presence of cell proliferation indicator Ki67 [82, 83]. The overexpression of FOXM1 was associated with the progression and agression of cervical squamous cell carcinomas by enhancing cell proliferation [82], promoting malignant cell migration and invasion [84].
	**KIF14**	Kinesin family member 14	2.91	1.18	1.05	A study reported the high levels of KIF14 expression in cervical carcinoma cell line C-33A [85]. KIF14 overexpression was also correlated with poor prognosis, tumor aggressiveness, lymph node metastasis and resistance to paclitaxel treatment [86].
	**KIF23**	Kinesin family member 23	2.38	1.39	0.83	A research showed an increase of KIF23 levels in preinvasive CIN 1 and invasive cervical cancer [87].
	**MAD2L1**	MAD2 mitotic arrest deficient-like 1 (yeast)	1.82	1.11	0.56	A study suggested that the significant overexpression of MAD2L1 in HSILs and SCCs may be involved in the cervical carcinogenesis [88].
	**MELK**	Maternal embryonic leucine zipper kinase	2.97	1.32	1.26	The high MELK expression was associated with poor prognosis and advanced tumor stage (CIN3/CIS and invasive cancer) [89].
	**NETO2**	Neuropilin and tolloid like 2	1.77	1.08	0.68	A research showed that the NETO2 mRNA level was increased in 50% of cervical cancer samples [90].
	**DKC1**	Dyskerin pseudouridine synthase 1	1.63	0.66	0.60	DKC1 has not been described to be associated with cervical cancer.
	LSG1^*^	Large 60S subunit nuclear export GTPase 1	1.62	0.66	1.11	LSG1 has not been described to be associated with cervical cancer.
	**NUP155**	Nucleoporin 155	1.77	0.93	0.90	NUP155 has not been described to be associated with cervical cancer.
	**POLR2H**	RNA polymerase II subunit H	1.98	1.36	1.28	POLR2H has not been described to be associated with cervical cancer.
**Gene dosage alterations revealed by cDNA microarray analysis in cervical cancer: Identification of candidate amplified and overexpressed genes** **[91]**	**PRKCI**	Protein kinase C iota	1.75	0.85	0.95	PRKCI overexpression was frequently observed in cervical squamous cell carcinoma [92].
	**RAD1**	RAD1 checkpoint DNA exonuclease	1.34	1.26	0.54	A research suggested that the mutation in pathways containing RAD1 may predispose to cervical cancer transition [93].
	RFC4^*^	Replication factor C subunit 4	3.03	1.55	1.23	The upregulation of RFC4 was observed in cervical cancer [91]. A study found that RFC4 may be used as a predictor of cancer relapse and survival rate in patients with cervical carcinoma [94].
	ZNF473^*^	Zinc finger protein 473	1.28	0.90	0.62	ZNF473 has not been described to be associated with cervical cancer.

^*^ The gene of which its expression pattern is associated with the prognosis in TCGA cohort (*P*-value < 0.05).

### Class prediction using deep learning and other machine learning algorithms

**Figure 3:**
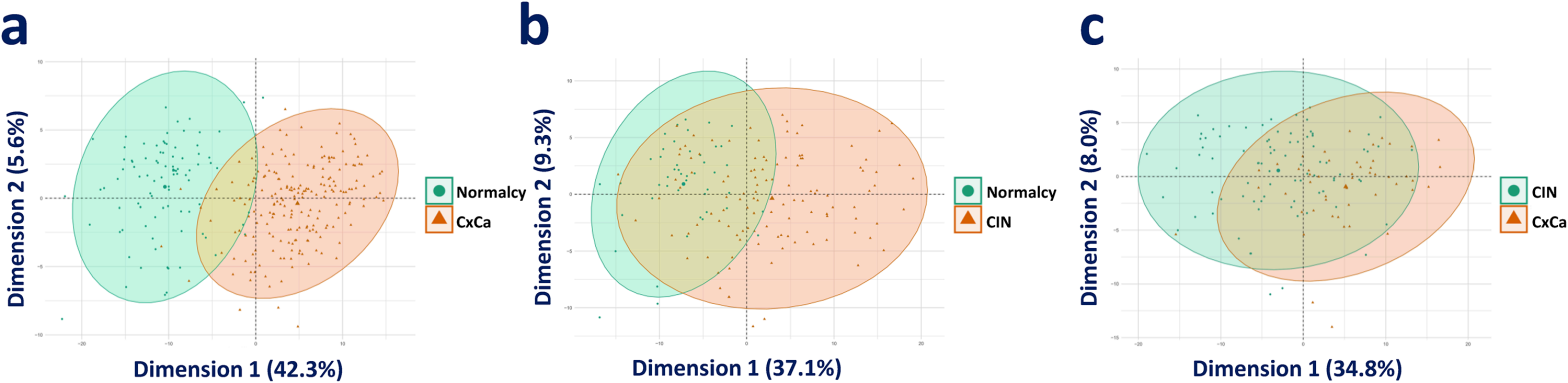
PCA visualization of the comparison groups **(a)** PCA plot of Cancer versus Normalcy. **(b)** PCA plot of CIN versus Normalcy. **(c)** PCA plot of Cancer versus CIN.

The consistently upregulated and downregulated genes in the meta-analysis of the three comparison groups (Cancer versus Normalcy, CIN versus Normalcy, and Cancer versus CIN) were selected as a genetic panel for unsupervised data exploration and deep learning classification analysis. Principal component analysis (PCA) revealed significantly overlapping areas of CIN versus Normalcy and Cancer versus CIN groups, which suggested that those classes were largely convergent and difficult to be correctly classified. On the other hand, the separation tendency between Cancer and Normalcy was relatively clear, implying a preferable classification. The PCA plots of Cancer versus Normalcy, Cancer versus CIN, and CIN versus Normalcy groups are shown in Figure 3a, Figure 3b, and Figure 3c, respectively.

**Figure 4:**
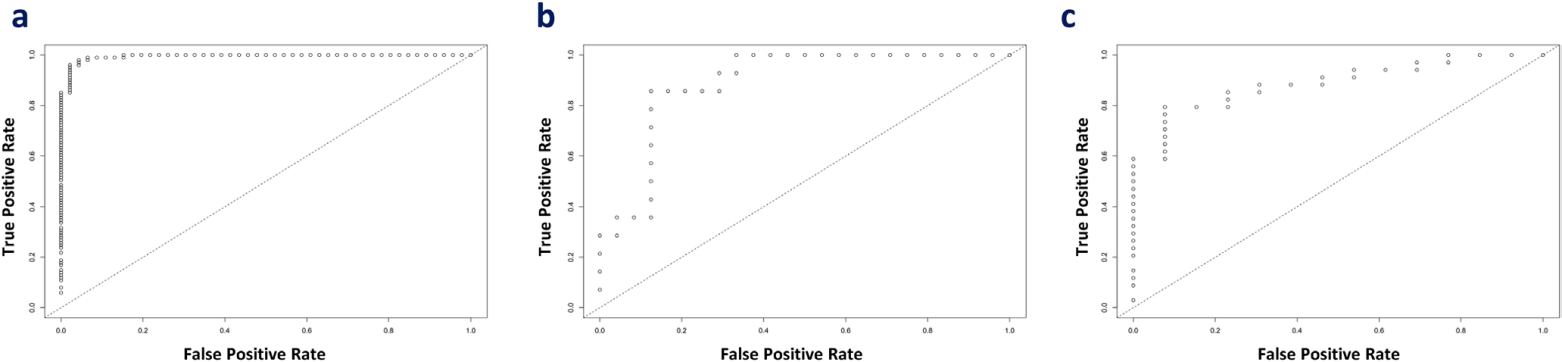
ROC curve for the illustration of the diagnostic ability of the panel **(a)** Cancer versus Normalcy, **(b)** Cancer versus CIN, and **(c)** CIN versus Normalcy.

In Cancer versus Normalcy group, the random search for hyperparameters revealed that an optimal model had five hidden layers, l1 regularization of 1.71E-4, and l2 regularization of 1.75E-4 (5, 1.71E-4, 1.75E-4). Similarly, the optimal models of Cancer versus CIN and CIN versus Normalcy were determined to be (5, 6.27E-4, 3.78E-4) and (2, 7.17E-4, 7.75E-4), respectively. Receiver operating characteristic (ROC) curves of the diagnostic ability of the three above two-class classifications are illustrated in Figure 4. The results indicated that deep learning models could distinctly differentiate the benign from malignant conditions. However, it can be argued that the use of the 168-gene signature for classification may show over-optimistic results since the signature was extracted by the meta-analysis using partly overlapping data sets. Hence, to search for a generalized and data-driven classification signature, we first performed the variable important measurement using the area under the curve permutation random forest variable importance measurement (AUC-RF VIM) on the training sets, selected the top 30 genes with the highest importance scores, and then built three new deep learning models. Supplementary File 5 contains the list of selected genes for the classification experiments, and they are markedly different among the three comparison groups. As expected, the new models achieved results comparable with the models derived from the 168-gene signature, and these classification models accurately discriminated the Cancer from CIN, Cancer from Normalcy, and CIN from Normalcy groups. The accuracy, sensitivity, and specificity of training sets with 10-fold cross-validation (combined holdout predictions, calculated from the confusion matrix) and test sets can be found in Table 5.

**Table 5:** Sensitivity and Specificity of deep learning classifier of three comparison groups

Group	Parameters	10-fold cross-validation	Test set
*168-gene signature*			
Cancer versus Normalcy			
	Accuracy (%)	97.97	97.96
	Sensitivity (%)	97.03	99.01
	Specificity (%)	100.00	95.65
Cancer versus CIN			
	Accuracy (%)	84.04	84.21
	Sensitivity (%)	71.43	85.71
	Specificity (%)	91.53	83.33
CIN versus Normalcy			
	Accuracy (%)	90.35	91.49
	Sensitivity (%)	91.36	91.18
	Specificity (%)	87.88	92.31
***AUC-RF VIM-based signatures***			
Cancer versus Normalcy			
	Accuracy (%)	97.30	97.28
	Sensitivity (%)	98.02	99.01
	Specificity (%)	95.74	93.48
Cancer versus CIN			
	Accuracy (%)	92.55	86.84
	Sensitivity (%)	91.43	85.71
	Specificity (%)	93.22	87.50
CIN versus Normalcy			
	Accuracy (%)	89.47	82.98
	Sensitivity (%)	90.12	88.24
	Specificity (%)	87.88	69.23

Finally, for a quick assessment of the class prediction of the ensemble model that combined four other powerful classifiers (random forest, support vector machine, prediction analysis for microarrays, and k-nearest neighbor algorithms), we performed the classification analyses for the three comparison groups (Cancer versus Normalcy, Cancer versus CIN, and CIN versus Normalcy) with whole transcriptome data. Regarding Cancer versus Normalcy group, we possessed a model from 40 frequently selected genes for distinguishing cancer patients from normal people (sensitivity = 88.2% and specificity = 95%), with an average accuracy of 92.8%. For discriminating Cancer from CIN patients, we achieved a model from 68 frequently selected genes (sensitivity = 85.5% and specificity = 71.4%), with an average accuracy of 80.3%. Finally, the differentiation between CIN and Normalcy group was practical since the prediction model from 54 frequently selected genes achieved a sensitivity = 73.9%, specificity = 84.3%, and average accuracy = 81.4%. Supplementary File 6 contains the list of selected genes, PCA of gene expression data, and heatmap visualization of top-ranked genes and their corresponding z-scores. Collectively, our results noted that the classification between the benign and the malignant conditions using small subsets of genes were practical.

### Prognostic assessment of the consistently up- and downregulated genes

**Figure 5:**
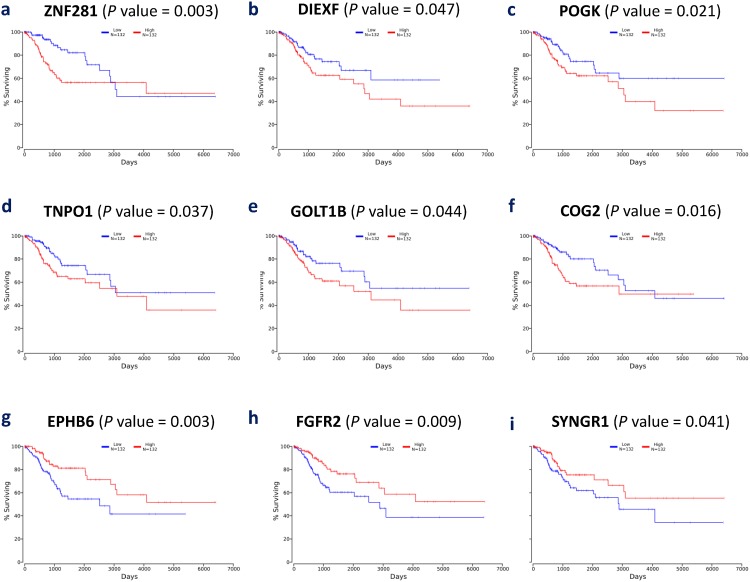
Kaplan-Meier plots of nine selected genes **(a)** ZNF281, **(b)** DIEXF, **(c)** POGK, **(d)** TNPO1, **(e)** GOLT1B, **(f)** COG2, **(g)** EPHB6, **(h)** FGFR2, and **(i)** SYNGR1.

**Figure 6:**
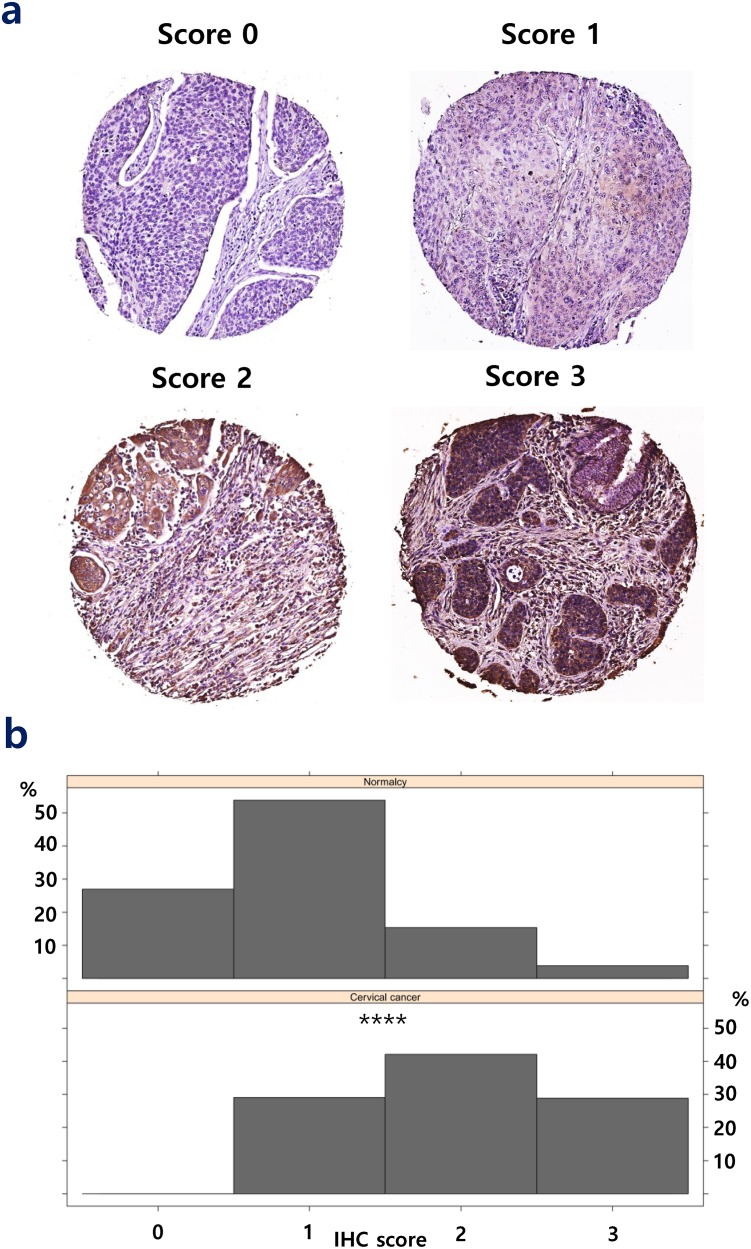
Protein expression level of ZNF281 in cancer samples **(a)** The representative staining scores (0-3) of ZNF281 in cancer tissues. **(b)** The staining score of ZNF281 is significantly higher in CxCa than in normal controls.^****^, *P*-value < 0.0001.

From the list of 113 consistently upregulated and 55 consistently downregulated genes, we performed a survival analysis to find individual genes that may be associated with the prognosis of CxCa patients. The prognostic assessment was conducted using TCGA cohort. It turned out that the higher gene expression of six genes, ZNF281, DIEXF, POGK, TNPO1, GOLT1B, and COG2, among consistently upregulated genes and the lower gene expression of three genes, SYNGR1, FGFR2, and EPHB6, among consistently downregulated genes, were associated with poor patient prognosis (Figure 5). These mentioned genes may be considered to be associated with the advanced stage of CxCa, as well as potential biomarker candidates to improve the prognostic assessment. In addition, Cox regression analysis revealed that ZNF281 was the only candidate gene that appeared to be related to poor CxCa prognosis (Cox coefficient = 0.53, adjusted *P*-value < 0.1). Collectively, ZNF281 may be a key regulator of CxCa initiation and progression. It also exhibits a good potential for both diagnostic and prognostic assessment and as a therapeutic target. Therefore, we further assessed the protein expression level of ZNF281 using tissue microarray samples of an independent cohort of CxCa patients and normal controls. As shown in Figure 6, the protein expression level of ZNF is significantly higher in CxCa samples than that of normal samples (two-tailed *P*-value < 0.0001). On the other hand, EPHB6 gene expression level may become the potential candidate for good CxCa prognosis (Cox coefficient = -0.45, adjusted *P*-value < 0.1).

## DISCUSSION

One of the main purposes of this study was to investigate the relevant biological processes specifically related to the initiation, invasion, and progression of CxCa with better confidence compared to individual studies through an integrative systems biology approach. Therefore, we first performed a random effect model-based meta-analysis to detect DE genes with a higher robust level. Functional analysis of the DE genes of each comparison group revealed that there were many significantly enriched pathways when a normal cell becomes cancerous. Interestingly, cell cycle, mRNA surveillance, and RNA transport pathways were always significantly enriched in the more malignant stages of CxCa carcinogenesis (from Normalcy to CIN and from CIN to Cancer). These results suggest that these pathways perturbations are strongly correlated with CxCa initiation and progression. Notably, cell cycle has a known central role in cancer carcinogenesis and metastasis [19, 20]. Cell cycle-related genes, including CDKN2A and MCM2, are overexpressed in HPV (+) CxCa. p16^INK4a^ (a product protein of CDKN2A) overexpression has been known to be associated with high grade precancerous and CxCa lesions and used as a potential biomarker for identifying low-grade lesions associated with high risk carcinomagenesis [21, 22]. However, a recent meta-analysis showed that the overexpression of p16^INK4a^ was associated with better prognosis of CxCa patients [23]. Other studies demonstrated that HPV infection alters the cell cycle and promotes cervical oncogenesis [24–27]. It is also worth mentioning that cell cycle-associated genes are significantly upregulated more in HPV (+) CxCa than in HPV (-) CxCa [28]. The upregulation of a cell cycle subnetwork with highly frequent alterations of its regulatory genes in CxCa has also been described [27]. Additionally, lncRNA Hox transcript antisense intergenic RNA (HOTAIR) promotes metastasis by increasing cell proliferation, migration, and the invasion of cancer cells, and the CxCa patients with high levels of HOTAIR often have a poorer prognosis. The pathway analysis of the DE genes (with logarithm fold change > 2) using GEO2R of GSE67522 revealed that the cell cycle is also significantly enriched by the contribution of CDC6, RBX1, SKP1, CCNE1, CDK2, CUL1, and MYC. Using network-based meta-analysis, we identified HDAC1 of the cell cycle pathway as the prominent hub gene in Cancer versus Normalcy and CIN versus Normalcy groups, while CREBBP was the prominent hub gene, among others, in Cancer versus CIN group. HDAC1 was reported to be more upregulated in cervical dysplasia and carcinoma than in normal cells [29]. The overexpression of HDAC1 was also associated with cell proliferation in breast cancer, cancer stem cells, and prostate cancer [30–33]. Mutation of CREBBP was described in relapsed acute lymphoblastic leukemia [34]. However, our analysis revealed that the gene expression levels of HDAC and CREBBP were not correlated with the prognosis of CxCa patients in TCGA cohort.

Recently, the association between cell cycle and metabolism in the survival mechanism of cancer cells has been demonstrated [35]. Moreover, the one carbon pool by folate pathway, which was upregulated in Cancer versus Normalcy and CIN versus Normalcy groups in our study, has been noted as a predominant pathway in cancer cell survival and progression for many years [36]. TYMS, SHMT2, and DHFR genes of one carbon metabolism are proven to contribute to genome instability and cancer development [37]. MTHFD2, another gene associated with one carbon metabolism, is considered to be an important player for various types of cancer and correlated with mitochondrial folate metabolism [38]. Moreover, the important roles of MTHFD2 in cell proliferation, DNA synthesis control, and cell migration in cancer have been confirmed [39, 40]. Thus, the connection between cell cycle and one carbon metabolism in CxCa is an attractive target for prospective studies. RNA transport and mRNA surveillance are the two other major pathways of CxCa pathobiology in our study. The association between these pathways and viral genome replication in HPV (+) CxCa has been documented [41, 42]. The viral oncogenes and their proteins expressions (E5, E6, and E7, for example) increase the cell proliferation, inhibit apoptosis resulting from DNA damage, and enhance malignant transformation [43]. However, the fundamental regulatory mechanisms of RNA transport and mRNA surveillance associated genes remain to be explored.

Although the use of high-throughput gene expression profiling for disease diagnosis is promising, the clinical and translational potential of this approach has been limited due to the failure of cross-study validation [44]. Nevertheless, we extended the capacity of gene expression profiling on CxCa and CIN diagnosis by combining independent data sets using the Empirical Bayes cross-study normalization method and conducting deep learning classifications on the relatively large sample size of transcriptome data of CxCa, CIN, and normal samples. The results demonstrate that the patients with CxCa can be accurately diagnosed by genetic classification panels using deep learning techniques and by the ensemble supervised learning tools. Noticeably, the benign and the malignant conditions are differentiated just by a small subset of gene expression information. This differentiation may help improve the utility of the transcriptome-based diagnosis in clinical practice. In addition, gene expression patterns from tumors may be used for better patient management, as is the case, for example, in breast cancer. Further studies are guaranteed to improve the classification performance by either increasing the sample size, and not limiting the study to Affymetrix-based microarray data, or improving the predictive power of the supervised learning models.

In the prognostic assessment, ZNF281, DIEXF, POGK, TNPO1, GOLT1B, COG2, SYNGR1, FGFR2, and EPHB6 are found to be associated with the prognosis of CxCa patients. Notably, those genes are consistently up- and downregulated in a multi-stage manner; thus, they can be considered to be associated with tumor development and aggression. Nevertheless, the precise roles of these genes in regulating CxCa remain unknown. DIEXF is known as an alternative polyadenylation-indicator gene in non-small cell lung cancer, and its expression is correlated with cell proliferation [45]. A study indicated the presence of a TNPO1–IKBKB (IKK-beta) fusion in prostate cancer but not in benign tissue [46]. The shortening of 3′ UTRs of SYNGR1 was associated with poorer prognosis in triple-negative breast cancer [47]. FGFR2 was involved in increased risk of breast cancer [48]. To the best of our knowledge, the roles of POGK, GOLT1B, and COG2 in carcinogenesis have not been identified.

Among the candidates, the dysregulation of ZNF281 at the protein level in CxCa was confirmed in our study. Zinc finger proteins play a central role in diverse biological processes, including monitoring gene expression. The regulation mechanisms of those proteins in cancer progression vary among different types of cancer or even in the same cancer at different stages [49]. ZNF281, a zinc finger transcription factor, belongs to the C2H2-type zinc finger motif, a subfamily of zinc finger proteins. Although the known functions of ZNF281 in cancer biology have been limited, recent studies have provided new insights into the function of ZNF281 in EMT and its association with the WNT signaling pathway [50, 51]. ZNF281 can serve as an EMT-inducting transcription factor (EMT-TF), along with SNAIL, SLUG, TWIST1/2, and ZEB1/2, which induces EMT transcriptional changes. Furthermore, the expression of ZNF281 is directed by SNAIL, an EMT inducer, and in turn, required for the transcription of SNAIL. In addition, ZNF281 itself regulates a number of EMT-related effector genes [52]. The abovementioned evidence combined with our findings suggest that ZNF281 may be considered as a new prognostic and therapeutic target for the management of CxCa. However, the precise role of ZNF281 in CxCa initiation and progression remains to be elucidated.

Our study has several limitations. Firstly, the distinct gene expression biosignatures of cervical adenocarcinoma (five samples) and cervical adenosquamous carcinoma (one sample) were unable to be explored. Secondly, we did not examine the effects of HPV to CxCa carcinogenesis, but instead, focused on the general process in the multi-stage manner. Finally, the combination of CIN1, CIN2, and CIN3 on behalf of cervical precancerous lesions may introduce biases since they are associated with different subtypes of HPV (CIN1 and CIN2 mainly with intermediate risk HPV genotypes while CIN 3 mainly with HPV16).

Better strategies for diagnosis and prognostic assessment of CxCa can be achieved by improving our understanding of the formation and development of the disease. Although the mechanisms of CxCa carcinogenesis, especially HPV integration-driven cervical carcinogenesis have been studied, the alterations of genes in the dysregulated processes and their effects have not been intensively scrutinized. In the current study, we conducted a comprehensive analysis and suggest new evidence on the mechanisms of cervical oncogenesis and metastasis. Our findings also indicated that the overexpression of cell cycle, one carbon pool by folate, RNA transport, and mRNA surveillance pathways play critical roles in the multi-stage development of CxCa. Genetic panels and a deep learning classification approach, which can be used to improve the diagnosis of CxCa, were proposed. We also introduced ZNF281 as a novel biomarker for the prognostic assessment of CxCa and a new potential therapeutic target. Other promising genes that may be significantly important to CxCa progression were also identified. In conclusion, the current study provides opportunities to improve current understanding of CxCa pathogenesis and hopefully helps improve CxCa patient outcome.

## MATERIALS AND METHODS

### Study design, search strategy, and selection criteria of the meta-analysis

A systematic search of Gene Expression Omnibus (GEO) and ArrayExpress was performed using the following query structure: “(“cervical” OR “cervix”) AND (“cancer” OR “cancers” OR “cancerous” OR “neoplasm” OR “neoplasms” OR “neoplastic” OR “tumor” OR “tumors” OR “tumour” OR “tumours” OR “tumorous” OR “tumourous”)”. Our results covered all available data sets published up to December 2016. Identified data sets were evaluated for eligibility by at least two authors. Disagreements among reviewers were resolved by consensus with other reviewers. A data set was included if it contained Affymetrix-based microarray data and the study design met the following criteria: the data set provided clear definitions of analyzed samples; the data set contained at least one comparison among Cancer versus Normalcy, Cancer versus CIN, or CIN versus Normalcy groups. We excluded data sets for the following reasons: no relevant item found; the study was carried out from a cohort without a control group; the results contained overlapping data in other data set(s); and the studied subject was not human. Only the largest data set was included among overlapping data sets. We also manually searched the reference lists of the original studies of included data sets to find other relevant data sets.

### Data pre-processing, gene expression meta-analysis, and pathway enrichment analysis

In this study, data preprocessing, gene expression analysis and meta-analysis, and functional analysis were conducted by the published protocol of NetworkAnalyst with minor modifications in data set production and data normalization processes [17]. To normalize gene expression data of included data sets, we applied a robust multi-array analysis method using the Bioconductor Affy package [53, 54]. In addition, Database for Annotation, Visualization, and Integrated Discovery (DAVID) online software was used to convert the gene labels to Entrez IDs for unsupported probe platforms from NetworkAnalyst prior to the analysis [55]. A random effects model was utilized for the meta-analysis. Kyoto Encyclopedia of Genes and Genomes (KEGG) annotation was applied for pathway enrichment analysis to detect corresponding biologically enriched pathways. Potential hub genes in network analysis were detected by betweenness centrality (BC) and degree centrality (DC) of the first-order network. Protein-protein networks of the consistently up- or downregulated genes were derived from the Search Tool for the Retrieval of Interacting Genes/Proteins (STRING) database version 10.5 [56]. In the protein-protein networks, known interactions from curated databases and experiments were presented along with predicted interactions (minimum required interaction score = 0.4). Functional enrichments were detected using KEGG and Gene Ontology (GO) biological process where applicable (false discovery rate < 0.05).

### Text mining for gene list interpretation

CCancer web-based software was applied to examine whether our reported gene list was significantly enriched in terms of the intersection with previously reported gene lists for other biological processes [57]. This approach is based on the hypothesis that significant similarities among gene/protein lists may indicate potentially be a good indicator of similarity in molecular mechanisms among corresponding biological processes.

### Data exploration and visualization

Principal component analysis (PCA) is an orthogonal transformation analysis that aims to reduce the dimension of the data but at the same time minimizes information loss. In this study, we applied PCA to examine the tendency of separation among samples that belong to different groups. A heatmap was also used to visualize the data of selected features. The analysis and visualization were performed using FactoMineR, factoextra 1.0.4.999, and MetaboAnalyst 3.0 [58–60]. A Venn diagram was produced using InteractiVenn [18].

### Variable importance measurement and class assignment analysis

The heterogeneity of the data sets from different platforms was adjusted using the Combat method to integrate different data sets covering the same biological condition into one unique data set [61]. The corresponding data sets were later split into training and test sets using the caret package version 6.0-76 [62]. For Cancer versus Normalcy group, the splitting ratio was 50:50 while the splitting ratio for Cancer versus CIN and CIN versus Normalcy groups was 70:30. Variable importance measurements of the training sets were conducted using the area under the curve permutation random forest variable importance measurement (AUC-RF VIM) of party package version 1.2-3 [63]. The top 30 genes of each data set were introduced to the deep learning-based classification independently with the 168-gene panel.

The deep learning analyses were executed using H2O package version 3.10.3.6 in R environment version 3.3.3 [64]. For training the predictive models, the adaptive learning rate method for stochastic gradient descent optimization was used as a default. We carried out the random hyperparameter search for the number of hidden layers, l1 regularization, and l2 regularization. The number of layers was set from two to five, and each layer contained 200 neurons. The l1 regularization and l2 regularization searches were conducted using the sequence function containing the values from 0.00 to 1.00E-3 by 1.00E-6. The search criteria strategy was set as “Random Discrete” for searching all the combinations of the number of layers, l1 regularization, and l2 regularization. Other parameters were set empirically or by the default settings. The number of epochs was adjusted at 100. The Rectifier linear activation function was applied for a fast and accurate training process. Additional regularization methods, including dropout (hidden dropout ratios = 0.5) and early stopping (logloss stopping metric, stopping tolerance = 0.01, and stopping round = 5) were employed. The training sets were used for hyperparameter tuning processes via 10-fold cross-validation. The performances of the fine-tuned models with the optimal hyperparameters were then measured on the test sets. The cut-off of the classification was the corresponding value that optimize F1-score at default.

Ensemble class assignment analyses on the corresponding data sets of the whole transcriptome information were conducted using ArrayMining online software [65]. In brief, the data set was introduced to the Class Assignment Module with the settings of the ensemble method for feature selection. The ensemble method is a combination of three different univariate filters (Pearson correlation filter, signal-to-noise-ratio filter, and F-score filter) to make the ensemble feature ranking. An ensemble prediction method that combines random forest, support vector machine, prediction analysis for microarrays, and k-nearest neighbor algorithms were applied. This combination is supposed to provide a more robust classification analysis. For model evaluation, we performed 10-fold cross-validation. The maximum feature subset size was set at the default (30 features). The performance of the prediction models was appraised using accuracy, sensitivity, and specificity.

### Prognostic analysis

We investigated the prognostic ability of selected genes using Cancer Genome Atlas (TCGA) data set. mRNA-based Cox regression analysis of a cohort of 264 TCGA patients were extracted using Oncolnc [66]. Cox regression factors on survival were set at default: “coxph(Surv(times, died) ∼ gene + grade1 + grade2 + grade3 + grade4 + age)”. In addition, we exploited the Kaplan-Meier method with log-rank test for comparing survival curves in two groups with the divided option of upper 50 percent mRNA expression and lower 50 percent mRNA expression patients. A log-rank *P*-value less than 0.05 (Kaplan-Meier) and adjusted *P*-value less than 0.1 (Cox regression) were considered to be the statistically significant thresholds.

### Tissue microarray (TMA) staining and analyses

Human cervical tissue microarray slides (CR6161) were purchased from the US Biomax Inc. (Rockville, MD, USA). Rabbit anti-ZNF281 (1:30, Atlas Antibodies, HPA051228) antibody was used for the experiment. Immunohistochemistry (IHC) was performed for human normal tissues and cervical cancer tissues using the protocol previously described with minor modifications [67]. Images were obtained from Aperio Scanscope digital slide scanners and analyzed by the vendor’s software (Leica, Wetzlar, Germany). The staining score was given as 0, 1, 2, and 3 for the tissues with stainless cells, <10% stained cells, 10-50% stained cells, and > 50% stained cells, respectively. Tissue loss cores were excluded from the analysis. Only squamous cell carcinoma, adjacent normal, and normal tissues were considered in the final statistical analysis (256 cancer and 26 normal tissues). A Mann-Whitney U test was conducted to compare differences between the normal group and CxCa group using GraphPad Prism 6 (San Diego, CA). IHC scoring visualization was performed using lattice package version 0.20-35 in R environment version 3.3.3 [68]. The significance level was set at 0.05.

## SUPPLEMENTARY MATERIALS















## Abbreviations

CxCa: Cervical cancer
CIN: Cervical intraepithelial neoplasia
LSIL: Low-grade squamous intraepithelial lesion
HSIL: High-grade squamous intraepithelial lesion
HPV: Human papillomavirus
TCGA: Cancer Genome Atlas
STRING: Search Tool for the Retrieval of Interacting Genes/Proteins
IHC: Immunohistochemistry
PCA: Principal component analysis
AUC-RF VIM: The area under the curve permutation random forest variable importance measurement
ROC: Receiver operating characteristic curve

## References

[1] Ferlay J, Soerjomataram I, Dikshit R, Eser S, Mathers C, Rebelo M, Parkin DM, Forman D, Bray F (2015). Cancer incidence and mortality worldwide: sources, methods and major patterns in GLOBOCAN 2012. Int J Cancer.

[2] Jemal A, Bray F, Center MM, Ferlay J, Ward E, Forman D (2011). Global cancer statistics. CA Cancer J Clin.

[3] Crosbie EJ, Einstein MH, Franceschi S, Kitchener HC Human papillomavirus and cervical cancer. Lancet.

[4] Burd EM (2003). Human papillomavirus and cervical cancer. Clin Microbiol Rev.

[5] Zeng Q, Chen J, Li Y, Werle KD, Zhao RX, Quan CS, Wang YS, Zhai YX, Wang JW, Youssef M, Cui R, Liang J, Genovese N (2017). LKB1 inhibits HPV-associated cancer progression by targeting cellular metabolism. Oncogene.

[6] Torre LA, Bray F, Siegel RL, Ferlay J, Lortet-Tieulent J, Jemal A (2015). Global cancer statistics, 2012. CA Cancer J Clin.

[7] Wit EM, Horenblas S (2014). Urological complications after treatment of cervical cancer. Nat Rev Urol.

[8] Zhu H, Wu J, Zhang W, Luo H, Shen Z, Cheng H, Zhu X (2016). PKM2 enhances chemosensitivity to cisplatin through interaction with the mTOR pathway in cervical cancer. Scientific Reports.

[9] Saslow D, Runowicz CD, Solomon D, Moscicki AB, Smith RA, Eyre HJ, Cohen C (2002). American Cancer Society guideline for the early detection of cervical neoplasia and cancer. CA Cancer J Clin.

[10] Thekkek N, Richards-Kortum R (2008). Optical imaging for cervical cancer detection: solutions for a continuing global problem. Nat Rev Cancer.

[11] Cheng Q, Lau WM, Chew SH, Ho TH, Tay SK, Hui KM (2002). Identification of molecular markers for the early detection of human squamous cell carcinoma of the uterine cervix. Br J Cancer.

[12] del Pino M, Svanholm-Barrie C, Torne A, Marimon L, Gaber J, Sagasta A, Persing DH, Ordi J (2015). mRNA biomarker detection in liquid-based cytology: a new approach in the prevention of cervical cancer. Mod Pathol.

[13] Noordhuis MG, Fehrmann RS, Wisman GB, Nijhuis ER, van Zanden JJ, Moerland PD, Ver Loren van Themaat E, Volders HH, Kok M, ten Hoor KA, Hollema H, de Vries EG, de Bock GH (2011). Involvement of the TGF-beta and beta-catenin pathways in pelvic lymph node metastasis in early-stage cervical cancer. Clin Cancer Res.

[14] Walsh CJ, Hu P, Batt J, Dos Santos CC (2015). Microarray meta-analysis and cross-platform normalization: integrative genomics for robust biomarker discovery. Microarrays (Basel).

[15] Shabalin AA, Tjelmeland H, Fan C, Perou CM, Nobel AB (2008). Merging two gene-expression studies via cross-platform normalization. Bioinformatics.

[16] Fischer M, Grossmann P, Padi M, DeCaprio JA (2016). Integration of TP53, DREAM, MMB-FOXM1 and RB-E2F target gene analyses identifies cell cycle gene regulatory networks. Nucleic Acids Res.

[17] Xia J, Gill EE, Hancock RE (2015). NetworkAnalyst for statistical, visual and network-based meta-analysis of gene expression data. Nat Protoc.

[18] Heberle H, Meirelles GV, da Silva FR, Telles GP, Minghim R (2015). InteractiVenn: a web-based tool for the analysis of sets through Venn diagrams. BMC Bioinformatics.

[19] Collins K, Jacks T, Pavletich NP (1997). The cell cycle and cancer. Proc Natl Acad Sci U S A.

[20] Kastan MB, Bartek J (2004). Cell-cycle checkpoints and cancer. Nature.

[21] Benevolo M, Mottolese M, Marandino F, Vocaturo G, Sindico R, Piperno G, Mariani L, Sperduti I, Canalini P, Donnorso RP, Vocaturo A (2006). Immunohistochemical expression of p16INK4a is predictive of HR-HPV infection in cervical low-grade lesions. Mod Pathol.

[22] Romagosa C, Simonetti S, Lopez-Vicente L, Mazo A, Lleonart ME, Castellvi J, Ramon y Cajal S (2011). p16Ink4a overexpression in cancer: a tumor suppressor gene associated with senescence and high-grade tumors. Oncogene.

[23] Lin J, Albers AE, Qin J, Kaufmann AM (2014). Prognostic significance of overexpressed p16INK4a in patients with cervical cancer: a meta-analysis. PLoS One.

[24] Shai A, Brake T, Somoza C, Lambert PF (2007). The human papillomavirus E6 oncogene dysregulates the cell cycle and contributes to cervical carcinogenesis through two independent activities. Cancer Res.

[25] Hebner CM, Laimins LA (2006). Human papillomaviruses: basic mechanisms of pathogenesis and oncogenicity. Rev Med Virol.

[26] Kim YT, Zhao M (2005). Aberrant cell cycle regulation in cervical carcinoma. Yonsei Med J.

[27] van Dam PA, van Dam PJ, Rolfo C, Giallombardo M, van Berckelaer C, Trinh XB, Altintas S, Huizing M, Papadimitriou K, Tjalma WA, van Laere S (2016). In silico pathway analysis in cervical carcinoma reveals potential new targets for treatment. Oncotarget.

[28] Pyeon D, Newton MA, Lambert PF, den Boon JA, Sengupta S, Marsit CJ, Woodworth CD, Connor JP, Haugen TH, Smith EM, Kelsey KT, Turek LP, Ahlquist P (2007). Fundamental differences in cell cycle deregulation in human papillomavirus-positive and human papillomavirus-negative head/neck and cervical cancers. Cancer Res.

[29] Lin Z, Bazzaro M, Wang MC, Chan KC, Peng S, Roden RB (2009). Combination of proteasome and HDAC inhibitors for uterine cervical cancer treatment. Clin Cancer Res.

[30] Kawai H, Li H, Avraham S, Jiang S, Avraham HK (2003). Overexpression of histone deacetylase HDAC1 modulates breast cancer progression by negative regulation of estrogen receptor alpha. Int J Cancer.

[31] Muller BM, Jana L, Kasajima A, Lehmann A, Prinzler J, Budczies J, Winzer KJ, Dietel M, Weichert W, Denkert C (2013). Differential expression of histone deacetylases HDAC1, 2 and 3 in human breast cancer—overexpression of HDAC2 and HDAC3 is associated with clinicopathological indicators of disease progression. BMC Cancer.

[32] Witt AE, Lee CW, Lee TI, Azzam DJ, Wang B, Caslini C, Petrocca F, Grosso J, Jones M, Cohick EB, Gropper AB, Wahlestedt C, Richardson AL (2017). Identification of a cancer stem cell-specific function for the histone deacetylases, HDAC1 and HDAC7, in breast and ovarian cancer. Oncogene.

[33] Glozak MA, Seto E (2007). Histone deacetylases and cancer. Oncogene.

[34] Mullighan CG, Zhang J, Kasper LH, Lerach S, Payne-Turner D, Phillips LA, Heatley SL, Holmfeldt L, Collins-Underwood JR, Ma J, Buetow KH, Pui CH, Baker SD (2011). CREBBP mutations in relapsed acute lymphoblastic leukaemia. Nature.

[35] Wang H, Nicolay BN, Chick JM, Gao X, Geng Y, Ren H, Gao H, Yang G, Williams JA, Suski JM, Keibler MA, Sicinska E, Gerdemann U (2017). The metabolic function of cyclin D3–CDK6 kinase in cancer cell survival. Nature.

[36] Locasale JW (2013). Serine, glycine and one-carbon units: cancer metabolism in full circle. Nat Rev Cancer.

[37] Yang M, Vousden KH (2016). Serine and one-carbon metabolism in cancer. Nat Rev Cancer.

[38] Nilsson R, Jain M, Madhusudhan N, Sheppard NG, Strittmatter L, Kampf C, Huang J, Asplund A, Mootha VK (2014). Metabolic enzyme expression highlights a key role for MTHFD2 and the mitochondrial folate pathway in cancer. Nature Communications.

[39] Gustafsson Sheppard N, Jarl L, Mahadessian D, Strittmatter L, Schmidt A, Madhusudan N, Tegnér J, Lundberg EK, Asplund A, Jain M, Nilsson R (2015). The folate-coupled enzyme MTHFD2 is a nuclear protein and promotes cell proliferation. Scientific Reports.

[40] Lehtinen L, Ketola K, Mäkelä R, Mpindi JP, Viitala M, Kallioniemi O, Iljin K (2013). High-throughput RNAi screening for novel modulators of vimentin expression identifies MTHFD2 as a regulator of breast cancer cell migration and invasion. Oncotarget.

[41] McCance DJ (2005). Human papillomaviruses and cell signaling. Sci STKE.

[42] Smith SP, Scarpini CG, Groves IJ, Odle RI, Coleman N (2016). Identification of host transcriptional networks showing concentration-dependent regulation by HPV16 E6 and E7 proteins in basal cervical squamous epithelial cells. Sci Rep.

[43] zur Hausen H (2002). Papillomaviruses and cancer: from basic studies to clinical application. Nat Rev Cancer.

[44] Kim S, Lin CW, Tseng GC (2016). MetaKTSP: a meta-analytic top scoring pair method for robust cross-study validation of omics prediction analysis. Bioinformatics.

[45] Ichinose J, Watanabe K, Sano A, Nagase T, Nakajima J, Fukayama M, Yatomi Y, Ohishi N, Takai D (2014). Alternative polyadenylation is associated with lower expression of PABPN1 and poor prognosis in non-small cell lung cancer. Cancer Sci.

[46] Pflueger D, Terry S, Sboner A, Habegger L, Esgueva R, Lin PC, Svensson MA, Kitabayashi N, Moss BJ, MacDonald TY, Cao X, Barrette T, Tewari AK (2011). Discovery of non-ETS gene fusions in human prostate cancer using next-generation RNA sequencing. Genome Res.

[47] Wang L, Hu X, Wang P, Shao ZM (2016). The 3'UTR signature defines a highly metastatic subgroup of triple-negative breast cancer. Oncotarget.

[48] Campbell TM, Castro MA, de Santiago I, Fletcher MN, Halim S, Prathalingam R, Ponder BA, Meyer KB (2016). FGFR2 risk SNPs confer breast cancer risk by augmenting oestrogen responsiveness. Carcinogenesis.

[49] Jen J, Wang YC (2016). Zinc finger proteins in cancer progression. J Biomed Sci.

[50] Law DJ, Du M, Law GL, Merchant JL (1999). ZBP-99 defines a conserved family of transcription factors and regulates ornithine decarboxylase gene expression. Biochem Biophys Res Commun.

[51] Hahn S, Hermeking H (2014). ZNF281/ZBP-99: a new player in epithelial-mesenchymal transition, stemness, and cancer. J Mol Med (Berl).

[52] Hahn S, Jackstadt R, Siemens H, Hünten S, Hermeking H (2013). SNAIL and miR-34a feed-forward regulation of ZNF281/ZBP99 promotes epithelial–mesenchymal transition. EMBO J.

[53] Irizarry RA, Hobbs B, Collin F, Beazer-Barclay YD, Antonellis KJ, Scherf U, Speed TP (2003). Exploration, normalization, and summaries of high density oligonucleotide array probe level data. Biostatistics.

[54] Gautier L, Cope L, Bolstad BM, Irizarry RA (2004). affy—analysis of Affymetrix GeneChip data at the probe level. Bioinformatics.

[55] Huang da W, Sherman BT, Lempicki RA (2009). Systematic and integrative analysis of large gene lists using DAVID bioinformatics resources. Nat Protoc.

[56] Szklarczyk D, Franceschini A, Wyder S, Forslund K, Heller D, Huerta-Cepas J, Simonovic M, Roth A, Santos A, Tsafou KP, Kuhn M, Bork P, Jensen LJ (2015). STRING v10: protein-protein interaction networks, integrated over the tree of life. Nucleic Acids Res.

[57] Dietmann S, Lee W, Wong P, Rodchenkov I, Antonov AV (2010). Cancer: a bird's eye view on gene lists reported in cancer-related studies. Nucleic Acids Res.

[58] Xia J, Wishart DS (2016). Using MetaboAnalyst 3.0 for comprehensive metabolomics data analysis. Curr Protoc Bioinformatics.

[59] Lê S, Josse J, Husson F (2008). FactoMineR: an R package for multivariate analysis. J Stat Softw.

[60] Kassambara A, Mundt F (2017). factoextra: Extract and Visualize the Results of Multivariate Data Analyses. R package version 1.0.4.999.

[61] Walker WL, Liao IH, Gilbert DL, Wong B, Pollard KS, McCulloch CE, Lit L, Sharp FR (2008). Empirical Bayes accomodation of batch-effects in microarray data using identical replicate reference samples: application to RNA expression profiling of blood from Duchenne muscular dystrophy patients. BMC Genomics.

[62] Kuhn M (2008). Building predictive models in R using the caret package. J Stat Softw.

[63] Janitza S, Strobl C, Boulesteix AL (2013). An AUC-based permutation variable importance measure for random forests. BMC Bioinformatics.

[64] The H2O.ai team (2017). h2o: R Interface for H2O. R package version 3.10.3.6.

[65] Glaab E, Garibaldi JM, Krasnogor N (2009). ArrayMining: a modular web-application for microarray analysis combining ensemble and consensus methods with cross-study normalization. BMC Bioinformatics.

[66] Anaya J (2016). Oncolnc: linking tcga survival data to mrnas, mirnas, and lncrnas. PeerJ Comput Sci.

[67] Kampf C, Olsson I, Ryberg U, Sjostedt E, Ponten F (2012). Production of tissue microarrays, immunohistochemistry staining and digitalization within the human protein atlas. J Vis Exp.

[68] Sarkar D Lattice: Multivariate Data Visualization with R.

[69] Medina-Martinez I, Barrón V, Roman-Bassaure E, Juárez-Torres E, Guardado-Estrada M, Espinosa AM, Bermudez M, Fernández F, Venegas-Vega C, Orozco L, Zenteno E, Kofman S, Berumen J (2014). Impact of gene dosage on gene expression, biological processes and survival in cervical cancer: a genome-wide follow-up study. PLoS One.

[70] den Boon JA, Pyeon D, Wang SS, Horswill M, Schiffman M, Sherman M, Zuna RE, Wang Z, Hewitt SM, Pearson R, Schott M, Chung L, He Q (2015). Molecular transitions from papillomavirus infection to cervical precancer and cancer: role of stromal estrogen receptor signaling. Proc Natl Acad Sci U S A.

[71] Pappa KI, Polyzos A, Jacob-Hirsch J, Amariglio N, Vlachos GD, Loutradis D, Anagnou NP (2015). Profiling of discrete gynecological cancers reveals novel transcriptional modules and common features shared by other cancer types and embryonic stem cells. PLoS One.

[72] Caffarel MM, Chattopadhyay A, Araujo AM, Bauer J, Scarpini CG, Coleman N (2013). Tissue transglutaminase mediates the pro-malignant effects of oncostatin M receptor over-expression in cervical squamous cell carcinoma. J Pathol.

[73] Scotto L, Narayan G, Nandula SV, Arias-Pulido H, Subramaniyam S, Schneider A, Kaufmann AM, Wright JD, Pothuri B, Mansukhani M, Murty VV (2008). Identification of copy number gain and overexpressed genes on chromosome arm 20q by an integrative genomic approach in cervical cancer: potential role in progression. Genes Chromosomes Cancer.

[74] Zhai Y, Kuick R, Nan B, Ota I, Weiss SJ, Trimble CL, Fearon ER, Cho KR (2007). Gene expression analysis of preinvasive and invasive cervical squamous cell carcinomas identifies HOXC10 as a key mediator of invasion. Cancer Res.

[75] Rosty C, Sheffer M, Tsafrir D, Stransky N, Tsafrir I, Peter M, de Cremoux P, de La Rochefordiere A, Salmon R, Dorval T, Thiery JP, Couturier J, Radvanyi F (2005). Identification of a proliferation gene cluster associated with HPV E6/E7 expression level and viral DNA load in invasive cervical carcinoma. Oncogene.

[76] Narayan G, Arias-Pulido H, Nandula SV, Basso K, Sugirtharaj DD, Vargas H, Mansukhani M, Villella J, Meyer L, Schneider A, Gissmann L, Durst M, Pothuri B (2004). Promoter hypermethylation of FANCF: disruption of Fanconi Anemia-BRCA pathway in cervical cancer. Cancer Res.

[77] Narayan G, Arias-Pulido H, Koul S, Vargas H, Zhang FF, Villella J, Schneider A, Terry MB, Mansukhani M, Murty VV (2003). Frequent promoter methylation of CDH1, DAPK, RARB, and HIC1 genes in carcinoma of cervix uteri: its relationship to clinical outcome. Mol Cancer.

[78] Zhao M, Kim YT, Yoon BS, Kim SW, Kang MH, Kim SH, Kim JH, Kim JW, Park YW (2006). Expression profiling of cyclin B1 and D1 in cervical carcinoma. Exp Oncol.

[79] Zhang W, Liu Y, Zhao N, Chen H, Qiao L, Zhao W, Chen JJ (2015). Role of Cdk1 in the p53-independent abrogation of the postmitotic checkpoint by human papillomavirus E6. J Virol.

[80] Vazquez-Mena O, Medina-Martinez I, Juarez-Torres E, Barron V, Espinosa A, Villegas-Sepulveda N, Gomez-Laguna L, Nieto-Martinez K, Orozco L, Roman-Basaure E, Munoz Cortez S, Borges Ibanez M, Venegas-Vega C (2012). Amplified genes may be overexpressed, unchanged, or downregulated in cervical cancer cell lines. PLoS One.

[81] Mo W, Tong C, Zhang Y, Lu H (2015). microRNAs' differential regulations mediate the progress of Human Papillomavirus (HPV)-induced cervical intraepithelial neoplasia (CIN). BMC Syst Biol.

[82] Chan DW, Yu SY, Chiu PM, Yao KM, Liu VW, Cheung AN, Ngan HY (2008). Over-expression of FOXM1 transcription factor is associated with cervical cancer progression and pathogenesis. J Pathol.

[83] Guan P, Chen H, Li HJ, Duan J, Chen JY (2011). Expression and significance of FOXM1 in human cervical cancer: a tissue micro-array study. Clin Invest Med.

[84] He SY, Shen HW, Xu L, Zhao XH, Yuan L, Niu G, You ZS, Yao SZ (2012). FOXM1 promotes tumor cell invasion and correlates with poor prognosis in early-stage cervical cancer. Gynecol Oncol.

[85] Corson TW, Huang A, Tsao MS, Gallie BL (2005). KIF14 is a candidate oncogene in the 1q minimal region of genomic gain in multiple cancers. Oncogene.

[86] Wang W, Shi Y, Li J, Cui W, Yang B (2016). Up-regulation of KIF14 is a predictor of poor survival and a novel prognostic biomarker of chemoresistance to paclitaxel treatment in cervical cancer. Biosci Rep.

[87] Gius D, Funk MC, Chuang EY, Feng S, Huettner PC, Nguyen L, Bradbury CM, Mishra M, Gao S, Buttin BM, Cohn DE, Powell MA, Horowitz NS (2007). Profiling microdissected epithelium and stroma to model genomic signatures for cervical carcinogenesis accommodating for covariates. Cancer Res.

[88] Kim Y, Choi JW, Lee JH, Kim YS (2014). MAD2 and CDC20 are upregulated in high-grade squamous intraepithelial lesions and squamous cell carcinomas of the uterine cervix. Int J Gynecol Pathol.

[89] Rajkumar T, Sabitha K, Vijayalakshmi N, Shirley S, Bose MV, Gopal G, Selvaluxmy G (2011). Identification and validation of genes involved in cervical tumourigenesis. BMC Cancer.

[90] Oparina N, Sadritdinova AF, Snezhkina AV, Dmitriev AA, Krasnov GS, Senchenko VN, Mel'nikova NV, Belenikin MS, Lakunina VA, Veselovskii VA, Stepanov OA, Kudriavtseva AV (2012). Increase in NETO2 gene expression is a potential molecular genetic marker in renal and lung cancers. Genetika.

[91] Narayan G, Bourdon V, Chaganti S, Arias-Pulido H, Nandula SV, Rao PH, Gissmann L, Durst M, Schneider A, Pothuri B, Mansukhani M, Basso K, Chaganti RS (2007). Gene dosage alterations revealed by cDNA microarray analysis in cervical cancer: identification of candidate amplified and overexpressed genes. Genes Chromosomes Cancer.

[92] Regala RP, Thompson EA, Fields AP (2008). Atypical protein kinase C iota expression and aurothiomalate sensitivity in human lung cancer cells. Cancer Res.

[93] Scotto L, Narayan G, Nandula SV, Subramaniyam S, Kaufmann AM, Wright JD, Pothuri B, Mansukhani M, Schneider A, Arias-Pulido H, Murty VV (2008). Integrative genomics analysis of chromosome 5p gain in cervical cancer reveals target over-expressed genes, including Drosha. Mol Cancer.

[94] Huang L, Zheng M, Zhou QM, Zhang MY, Yu YH, Yun JP, Wang HY (2012). Identification of a 7-gene signature that predicts relapse and survival for early stage patients with cervical carcinoma. Med Oncol.

