# Supplementary material for: Systematic assessment of cervical cancer initiation and progression uncovers genetic panels for deep learning-based early diagnosis and proposes novel diagnostic and prognostic biomarkers
Source: Oncotarget. 2017 Nov 25;8(65):109436–56. doi: 10.18632/oncotarget.22689 (PMC5752532; doi:10.18632/oncotarget.22689)
Supplement: Supplementary file 1 [file oncotarget-08-109436-s001.pdf]

Systematic assessment of cervical cancer initiation and progression uncovers genetic panels for deep learning-based early diagnosis and proposes novel diagnostic and prognostic biomarkers

SUPPLEMENTARY MATERIALS

Supplementary File 1: The list of significantly DE genes of Cancer versus Normalcy, CIN versus Normalcy, and Cancer versus CIN groups, with their combined effect size

See Supplementary File 1

Supplementary File 2: The list of potential hub genes with their degree of centrality and betweenness centrality

See Supplementary File 2

Supplementary File 3: (a) The list of significantly enriched pathways and their corresponding genes in the three comparison groups analyzed from the list of upregulated DE genes. (b) The list of significantly enriched pathways and their corresponding genes of different comparison group analyzed from the list of downregulated DE genes

See Supplementary File 3

Supplementary File 4: The text mining results showing that the selected up- and downregulated genes were significantly enriched in previous cancer-related studies

See Supplementary File 4

Supplementary File 5: Top 30 important genes of Cancer versus Normalcy, Cancer versus CIN, and CIN versus Normalcy groups for the deep learning classification

See Supplementary File 5

Supplementary File 6: Sensitivity, specificity, average accuracy, z-score, and heatmap of the most frequently selected genes

See Supplementary File 6
